# Decreased serum level of thioredoxin 1 in female patients with pneumonia and its combinational use with haptoglobin for the specific diagnoses of pneumonia and lung cancer

**DOI:** 10.15172/pneu.2015.6/542

**Published:** 2015-12-01

**Authors:** Mee-Kyung Cha, Il-Han Kim

**Affiliations:** Department of Life Science and Technology, Pai Chai University, 11-3 Techno 1ro Yuseong-Gu, Daejeon, 305-509 Korea

**Keywords:** pneumonia, lung cancer, thioredoxin 1, haptoglobin, companion marker

## Abstract

Thioredoxin 1 (Trx1) and haptoglobin (Hp) are known to be involved in pathophysiology. This study was conducted to evaluate their diagnostic significance. We employed an enzyme-linked immunosorbent assay (ELISA) to determine the concentrations of both Trx1 and Hp in sera from female patients with community-acquired pneumonia (CAP) and those with lung cancer. The Trx1 levels remarkably decreased in cases of female patients with CAP, while the Hp levels increased in both female patients with lung cancer and CAP. In addition, the serum levels of Trx1 were not significantly changed in patients with lung cancer, rheumatoid arthritis, and cardiovascular diseases compared to healthy controls. At the cut-off point of 0.396 at A_450 nm_ on the receiver operating characteristic (ROC) curve, Trx1 could discriminate between patients with CAP from normal female controls with a sensitivity of 72.5%, a specificity of 89.8%, and area under the ROC curve (AUC) of 0.877 ± 0.040. The serum levels of Trx1 in female CAP patients were inversely correlated with the levels of Hp (*p* < 0.05). The characteristic reduction in serum Trx1 levels, especially in female CAP patients, indicates that Trx1 could be used as a diagnostic marker for CAP. The advantage of serum Trx1 over Hp in discriminating female CAP patients among female patients who have a positive serum level of Hp suggests the use of Trx1 as an excellent combination marker with Hp for the specific diagnosis of CAP and lung carcinoma, because serum Hp levels increase in female patients with lung cancer and those with CAP without selectivity.

## 1. Introduction

The 12-kDa human cytosolic thioredoxin 1 (Trx1) contains a redox-active dithiol moiety in its conserved active-site sequence (-Cys-Gly-Pro-Cys-) [1]. In resting cells, Trxl resides in the cytosol, but activation by a wide variety of stimuli leads to its translocation to the nucleus or the secretion of this protein [2,3]. Trxl catalyses thiol-disulfide exchange reactions via the two cysteine residues [1]. The exchange reaction activity is involved in various biological activities, including the regulation of the activity of transcription factors, such as nuclear factor (NF-ΚB) and activator protein-1 [2,4]. Moreover, reduced Trxl inhibits apoptosis by binding to apoptosis signaling kinase [1,5]. The extracellular Trx1 acts as a chemokine and co-cytokine, stimulating cytokine secretion and cell proliferation [6,7]. Due to these biological activities, Trx1’s involvement in redox processes could be important in the pathophysiology of cancer and inflammation [5–8].

Haptoglobin (Hp) protein, a plasma glycoprotein, is synthesised mainly in hepatocytes, dermal cells, pulmonary cells, and renal cells and released into the blood. In blood plasma, Hp binds free haemoglobin released from erythrocytes and prevents the loss of iron, and thereby inhibits haemoglobin’s oxidative activity [9]. Hp is also known as a positive acute-phase protein with a plasma level that rapidly increases in response to any infection or inflammatory process such as community-acquired pneumonia (CAP) [10–12]. In addition, elevated levels of Hp have been demonstrated in various cancers, including lung cancer [13–15].

CAP is the most common potentially fatal infectious disease worldwide [16]. Despite advances in prevention strategies, such as antibiotic treatment, significant improvement in the mortality rate is still lacking. This high mortality is mainly due to the absence of an effective diagnostic marker for CAP, which delays the timing of adequate antibiotic therapy. Because CAP is an infectious disease, commonly used laboratory values include the white blood cell count, C-reactive protein, and procalcitonin. In recent years, biomarkers have been intensively studied in CAP, not only for the correct diagnosis of CAP but also with respect to diagnosing its microbiological aetiology, the severity of disease, prognosis, and treatment decisions [17].

In the present study, we observed that the serum Trx1 levels are remarkably decreased in female patients with CAP compared to healthy female controls. We also demonstrated that the Hp level was significantly increased in serum from patients with CAP and those with lung cancer. The characteristic decrease of serum Trx1 levels, especially in female CAP patients, suggested that Trx1 could be useful as a diagnostic marker for CAP. Taken together, these findings indicated that Trx1 itself is a potential marker for CAP and is also an effective companion marker to Hp for the diagnoses of CAP and lung cancer.

## 2. Methods

### 2.1 Patients and sample collection

All sera of normal persons (control) and patients with CAP and lung cancer were obtained from Caucasians, White. The sera and the clinical information for the study participants were provided from Asterand Bioscience (https://doi.org/www.asterandbio.com) and BioServe (https://doi.org/www.bioserve.com) and are summarised in Table 1 and Table 2. All samples were collected from their collaborating clinical sites with full adherence to proper informed consent, as well as their strict institutional review board and *Health Insurance Portability and Accountability Act* compliance. To make them suitable for a biomarker study, all the sera were collected and treated according to the instructions of the Food and Drug Administration and the National Cancer Institute. All serum samples were taken prior to surgery and/or the commencement of chemotherapeutic medications. Samples were provided from subjects who were determined to have cardiovascular disease if they met the New York Heart Association criteria [18]; however, if the information for classification was not available, other diagnostic criteria of cardiovascular disease determined by echocardiogram, coronary angiogram, or high troponin levels following a cardiac episode were also included. Samples were provided from subjects with rheumatoid arthritis who met the American College of Rheumatology criteria for rheumatoid arthritis [19] and who were rheumatoid factor positive. Normal control serum samples were provided from subjects who were healthy and had no known clinical conditions (Eric M. Langlois, BioServe, Personal Communication, 31 March 2015).

**Table 1:** Characteristics of normal control subjects and non-cancer groups

Characteristics	No. of samples	Age (years) mean ± SD	BMI mean ± SD (range)	No. Smoking (%)
Normal control: Caucasian, White	100	44.1 ± 14.8	27.1 ± 4.6 (18.6–42.9)	54 (54.0)
Male	50	44.5 ± 14.9	27.5 ± 4.2 (18.6–38.2)	23 (46.0)
Female	50	44.2 ± 14.6	26.6 ± 4.9 (19.5–42.9)	31 (62.0)
CAP	80	67.0 ± 16.8	27.0 ± 5.9 (16.4–47.2)	56 (70.0)
Male	40	67.4 ± 16.6	25.8 ± 4.5 (16.4–37.9)	34 (85.0)
Female	40	66.7 ± 17.2	28.2 ± 6.8 (18.4–47.2)	22 (55.0)
Rheumatoid arthritis	30			
Female		59.5 ± 13.1	28.9 ± 5.5 (17.2–42.2)	15 (50.0)
Cardiovascular disease	30			
Female		65.0 ± 11.3	30.8 ± 6.9 (18.8–51.1)	16 (53.3)

CAP, community-acquired pneumonia; BMI, body mass index; SD, standard deviation of the mean

**Table 2:** Characteristics of serum samples from patients with lung cancer

Characterisics	No. of samples
Non-small cell lung cancer	111 (65.4 ± 10.4)^a^
Male	50 (65.8 ± 10.2)
Female	61 (61.8 ± 10.3)
Non-small cell lung cancer stage (sub-stage)	
Stage I (IA 19, IB 20)	39
Stage II (IIA 16, IIB 16)	32
Stage III (IIIA 21, IIIB 9)	30
Stage IV	10
Non-small cell lung cancer sub-type	
Adenocarcinoma, NOS	29 (19)^b^
Adenocarcinoma with mixed subtypes	17 (12)
Mixed cell adenocarcinoma	6 (3)
Papillary adenocarcinoma, NOS	2 (1)
Acinar cell carcinoma	7 (5)
Bronchiolo-alveolar adenocarcinoma, NOS	9 (2)
Bronchiolo-alveolar carcinoma, non-mucinous	1 (1)
Mucinous adenocarcinoma	5 (4)
Squamous cell carcinoma, NOS	19 (6)
Squamous cell carcinoma, keratinising, NOS	3 (2)
Adenosquamous carcinoma	3 (2)
Carcinoid tumor, NOS	2 (1)
Neuroendocrine carcinoma	2 (0)
N/A	6 (3)

There are no data on other tumor markers from the lung cancer samples.

NOS, not otherwise specified; N/A, not available

^a^Mean age ± SD, years; ^b^Number of female patients

### 2.2 Enzyme-linked immunosorbent assay (ELISA) for serum Trxl and Hp protein levels

An Express™ ELISA kit (rabbit), an indirect ELISA kit from GenScript Inc. (USA), was used to measure serum protein levels as per the manufacturer’s instructions. Antibodies to human Trx1 and Hp proteins were obtained by injecting purified proteins into rabbits to form antisera and then purifying the antiserum on a Protein A column. In brief, the sample dilution buffer containing an appropriate volume of human serum (5 µl for Trx1 determination; 10 µl for Hp determination) was coated onto a 96-well ELISA plate for 30 minutes, followed by the addition of the respective rabbit polyclonal antibodies (primary antibodies) against the antigens (Trx1 or Hp). After the primary antibody was allowed to react with the coated antigen for 1 hour, the antigen-antibody complex was treated with a horseradish peroxidase (HRP)-conjugated secondary antibody, followed by immobilisation for 1 hour and then washing 3 times with the washing solution. TMB (3, 3′, 5, 5′-tetramethylbenzidine) was used as the substrate, and a sulfuric acid solution (2 M sulfuric acid) was used to stop the enzymatic reaction. Protein levels were determined by measuring the absorbance at 450 nm in triplicate; mean values of 3 measurements are given. The intra-assay precision (coefficient of variations [CV]) for analysis of Trx1 and Hp were 5.77 ± 2.49% (mean ± SD) and 2.57 ± 1.23%, respectively. The interassay CV% was 9.18 ± 3.53% and 5.97 ± 3.97%, respectively, based on a sample of 30 female patients with CAP.

### 2.3 Statistical analysis

GraphPad Prism version 5.04 for Windows (GraphPad Software, USA) and MedCalc statistical software version 12.4.0.0 (MedCalc Software bvba, Belgium) were used for statistical analyses. We used the Pearson product-moment correlation coefficient to test for associations between different variables. The *t* tests and one-way analysis of variance (ANOVA) tests were performed to calculate the *p* value. The *p* value was considered to be statistically significant if *p* < 0.05. Testing for normality of the data was performed using the D’Agostino-Pearson normality test.

## 3. Results

### 3.1 Hp levels in lung cancer and CAP patients

**Figure 1:**
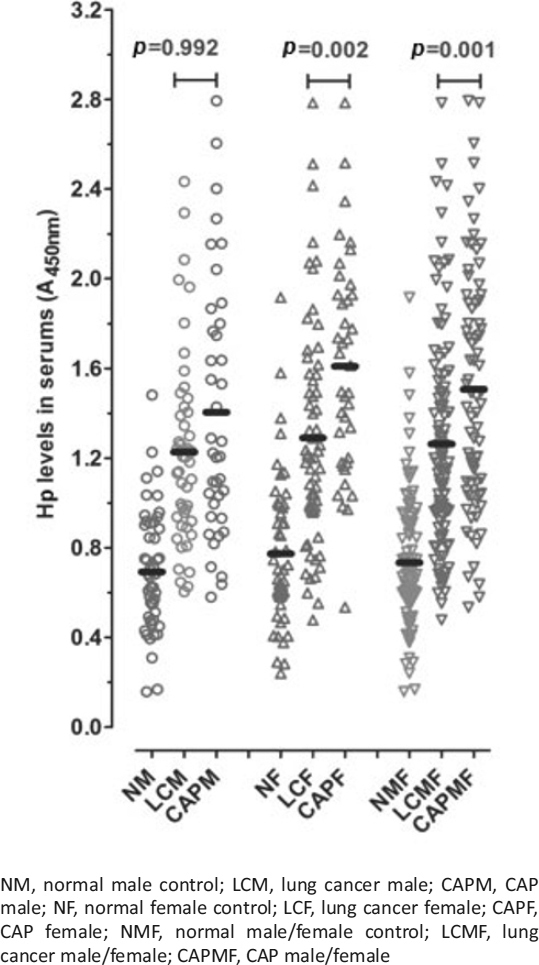
Serum haptoglobin (Hp) levels in the lung cancer and community-acquired pneumonia (CAP) groups. Clinicopathological information for each patient was provided by the supplier. The individual mean value (*n* = 3) is depicted as a scatter plot. The median value of each group is depicted by a horizontal line. The statistic values are shown in Table 3.

A potential difference in serum Hp levels between lung cancer and CAP patients was examined. In detail, serum Hp levels were measured in 111 lung cancer patients distributed over a wide range of ages (age distribution: 41 to 85 years) and 80 CAP patients (age distribution: 41 to 85 years) and subjected to receiver operating characteristic (ROC) curve plotting. Concentration measurements are graphically shown in Figure 1 and statistically summarised in Table 3. Area under the ROC curve (AUC) values were calculated by the partial integration of the ROC curve; sensitivity and specificity values are summarised in Table 4.

**Table 3:** Statistics for the serum haptogobulin (Hp) levels of normal control subjects, lung cancer, and community-acquired pneumonia (CAP) patients

	NM	LCM	CAPM	NF	LCF	CAPF	NMF	LCMF	CAPMF
Number of values	50	50	40	50	61	40	100	111	78
25% percentile	0.488	0.928	0.939	0.585	0.965	1.18	0.546	0.958	1.08
Median	0.676	1.20	1.22	0.702	1.20	1.61	0.682	1.20	1.46
75% percentile	0.904	1.42	1.80	0.984	1.58	1.93	0.914	1.49	1.90
Mean	0.693	1.23	1.40	0.774	1.29	1.61	0.733	1.26	1.51
Std. deviation	0.267	0.421	0.575	0.337	0.502	0.482	0.305	0.467	0.537
Std. error	0.038	0.060	0.092	0.048	0.064	0.077	0.031	0.045	0.061

NM, normal male control; LCM, lung cancer male; CAPM, CAP male; NF, normal female control; LCF, lung cancer female; CAPF, CAP female; NMF, normal male/female control; LCMF, lung cancer male/female; CAPMF, CAP male/female

**Table 4:** Parameters from the ROC analysis on the serum haptoglobin (Hp) levels in lung cancer and community-acquired pneumonia (CAP) patients

	Lung cancer			CAP		
	LCM	LCF	LCMF	CAPM	CAPF	CAPMF
AUC ± SE^a^	0.878 ± 0.033	0.820 ± 0.040	0.860 ± 0.028	0.865 ± 0.040	0.905 ± 0.037	0.882 ± 0.027
Sensitivity (%)	75.5	78.7	76.6	72.5	82.5	81.2
Specificity (%)	84.0	73.5	79.8	88.0	89.8	83.8
Cut-off value(A_450nm_)	0.938	0.954	0.954	0.958	1.136	0.995

Hp measurements of the normal male and female controls used as the reference

LCM, lung cancer male; LCF, lung cancer female; LCMF, lung cancer male/female; CAPM, CAP male; CAPF, CAP female; CAPMF, CAP male/female; AUC, area under the ROC curve; SE, standard error of the mean

^a^*p* < 0.0001

As seen in Figure 1 and Table 3, the mean Hp concentrations in the normal male/female control (NMF) group, the lung cancer male/female (LCMF) group, and the CAP male/female (CAPMF) group were 0.733, 1.26, and 1.51, respectively. This shows that the serum Hp levels increased by approximately 72% and 106% in LCMF and CAPMF patients, respectively, compared to NMF subjects, indicating that the Hp level is approximately 20% higher in CAP patients than in lung cancer patients. The difference in the elevation of Hp levels in the lung cancer versus CAP groups was more profound in the female groups (LCF versus CAPF:∼25%) than that in the male groups (LCM versus CAPM:∼14%). As indicated in Figure 1, the difference in the serum Hp level between male patients with lung cancer and CAP is not statistically significant (*p* > 0.05), whereas the difference between female patients is evident (*p* = 0.002).

For the LCM and CAPM groups compared to the NM subjects, the AUC values of Hp were measured to be, respectively, 0.878 and 0.865, with a sensitivity of 75.5% and 72.5% and a specificity of 84.0% and 88.0% (Table 4). For the LCF and CAPF groups compared to the NF subjects, AUC values of Hp were, respectively, 0.820 and 0.905, with a sensitivity of 78.7% and 82.5% and a specificity of 73.5% and 89.8%.

These data show that Hp can be used to screen for lung cancer and CAP, as displayed in Table 4 (AUC values for LCMF: 0.860, those for CAPMF: 0.882), although Hp has a disadvantage in its selectivity due to simultaneous elevations of Hp levels in both patient groups. It is worth noting that the selectivity between female lung cancer and CAP patients is superior compared to male patients.

### 3.2 Trx1 levels in patients with CAP and various diseases

We then examined the difference in serum Trx1 levels between patients with CAP and those with lung cancer. We aimed to investigate serum Trx1 levels due to reports that the intracellular synthesis of Trx1 increases in response to cancerous processes [5]. Serum Trx1 levels may also be increased in response to lung carcinoma progression. We selected lung cancer as a representative cancer for this study because CAP is an inflammatory disease of the lung. Serum Trx1 levels were measured by ELISA in the patients, as well as in normal male and female controls.

**Figure 2:**
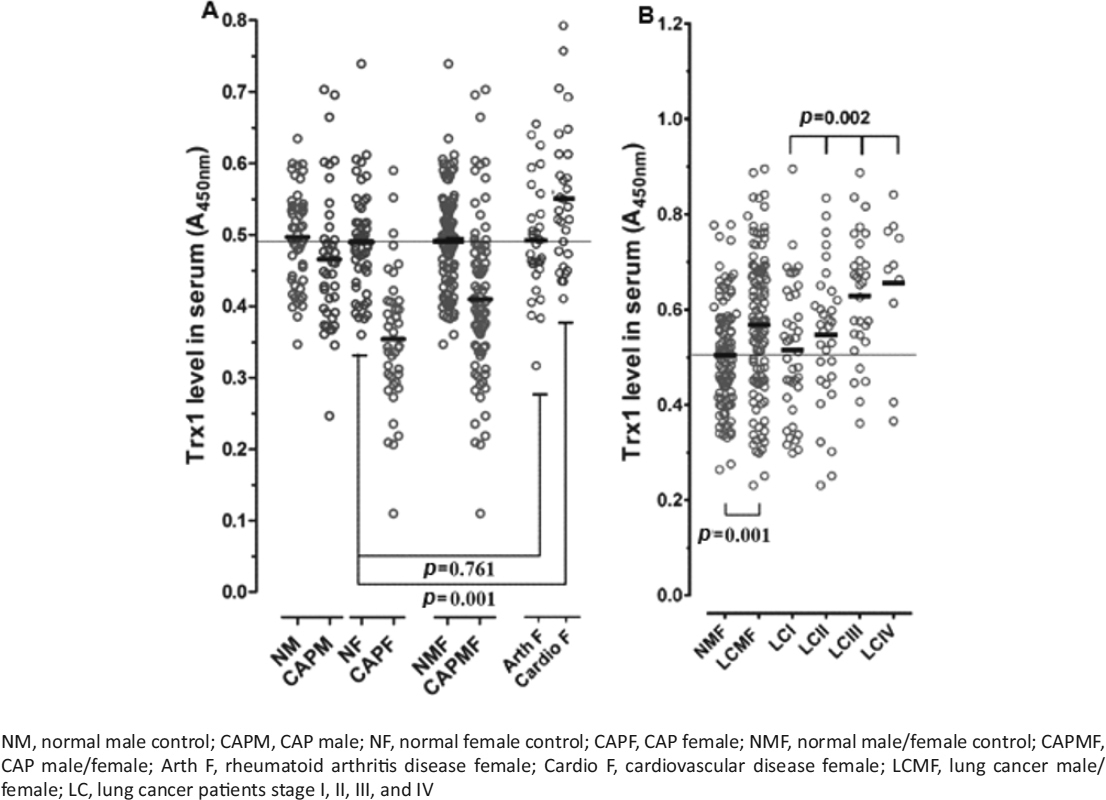
Serum thioredoxin 1 (Trx1) levels in the community-acquired pneumonia (CAP), rheumatoid arthritis, cardiovascular, and lung cancer groups. Changes of serum Trx1 levels along with lung cancer progression are depicted in panel B. The individual mean value (*n* = 3) is depicted as a scatter dot plot with the median value of each group (horizontal line). The statistic values are shown in Table 3. The *p* value shown in the upper portion of panel B was obtained from one-way ANOVA, and the other *p* values were obtained from *t*-tests.

As seen in Figure 2, the mean concentrations of Trx1 in the NMF, CAPMF, and LCMF groups were 0.504, 0.417, and 0.568, respectively, showing that the serum levels of Trx1 decreased by approximately 21% in the CAPMF patients. Trx1 levels increased by approximately 13% in the LCMF group compared to the NMF group. In addition, Figure 2B shows that serum Trx1 levels increased along with lung cancer progression, indicating that the increase of Trx1 levels may be caused by the cancerous process. The data for NF, NM, LCM, and LCF subjects are not separately shown here because of their similarity with the data for the NMF and LCMF groups.

Interestingly, serum Trx1 levels were at remarkably lower values in CAPF patients (0.360) than in CAPM patients (0.457), although the Trx1 levels are nearly the same in the respective controls. This finding indicated that the serum levels of Trx1 decrease by approximately 27% and 8% in CAPF and CAPM patients, respectively, compared to their corresponding normal controls (NF: 0.490; NM: 0.495) (Figure 2A).

Serum Trx1 levels were also measured in female patients with rheumatoid arthritis (Arth F) and cardiovascular disease (Cardio F). We chose to look at these groups because the intracellular synthesis of Trx1 is increased in response to oxidative stress, so we reasoned that serum Trx1 levels may also be increased in response to inflammatory and oxidative stress-induced diseases. As seen in Figure 2A, the mean serum Trx1 concentrations in the Arth F and Cardio F groups were 0.485 and 0.552, respectively, indicating that Trx1 levels in the Arth F patients are nearly the same as the levels in the NF group (*p* = 0.761). In contrast, the Trx1 levels increased by approximately 3% in Cardio F patients compared to the NF group (*p* = 0.001). The opposite trends observed for the serum Trx1 levels between CAP and the other investigated diseases support the utility of Trx1 as a diagnostic marker for CAP over Hp because serum Hp levels are significantly elevated in both patients with CAP and those with lung cancer.

Taken together, the data described above indicate that Trx1 may be useful as a promising CAP marker, especially for female subjects, because it is capable of clearly identifying CAP patients, whether they have been affected by cancer or another disease.

To clarify this result, ROC curve analysis was performed to compare the Trx1 levels of the CAP group and the lung cancer group, which was used as a representative cancerous disease of the same organ, with the Trx1 measurements of the normal male and female controls. For the CAPM and CAPF groups versus the NMF group, the AUC values for Trx1 were measured to be, respectively, 0.638 and 0.877 with a sensitivity of 65.0% and 72.5% and a specificity of 64.0% and 89.8%, respectively (Table 5). For the LCMF group compared to the NMF group, the AUC value of Trx1 was measured to be 0.636, with a sensitivity of 62.2% and a specificity of 64.0%.

**Table 5:** Parameters from the ROC analysis on the serum thioredoxin 1 (Trx1) levels in patients with lung cancer and community-acquired pneumonia (CAP)

	CAP			Lung cancer
	CAPM	CAPF	CAPMF	LCMF
AUC ± SE^a^	0.638 ± 0.062	0.877 ± 0.040	0.756 ± 0.039	0.636 ± 0.038
Sensitivity (%)	65.0	72.5	51.2	62.2
Specificity (%)	64.0	89.8	92.9	64.0
Cut-off value (A_450nm_)	0.479	0.396	0.397	0.532

Trx1 measurements of the normal male and female controls used as the reference

CAPM, CAP male; CAPF, CAP female; CAPMF, CAP male/female; LCMF, lung cancer male/female; AUC, area under the ROC curve; SE, standard error of the mean

^a^*p* < 0.000

In addition, ROC curve analysis was performed for the Trx1 levels of the cardiovascular group, with the increased Trx1 values compared to normal female values. For the Cardio F group compared to the NF group, the AUC value of Trx1 was measured to be 0.677 ± 0.063, indicating that this type of oxidative stress-related disease apparently increases Trx1 levels.

Taken together, the results described above indicate that Trx1 can discriminate between the female CAP group and the normal female controls at a probability of about as high as 88% and can be used as a CAP marker with superior sensitivity and specificity.

### 3.3 Reverse correlation of Trx1 levels with Hp levels

**Figure 3:**
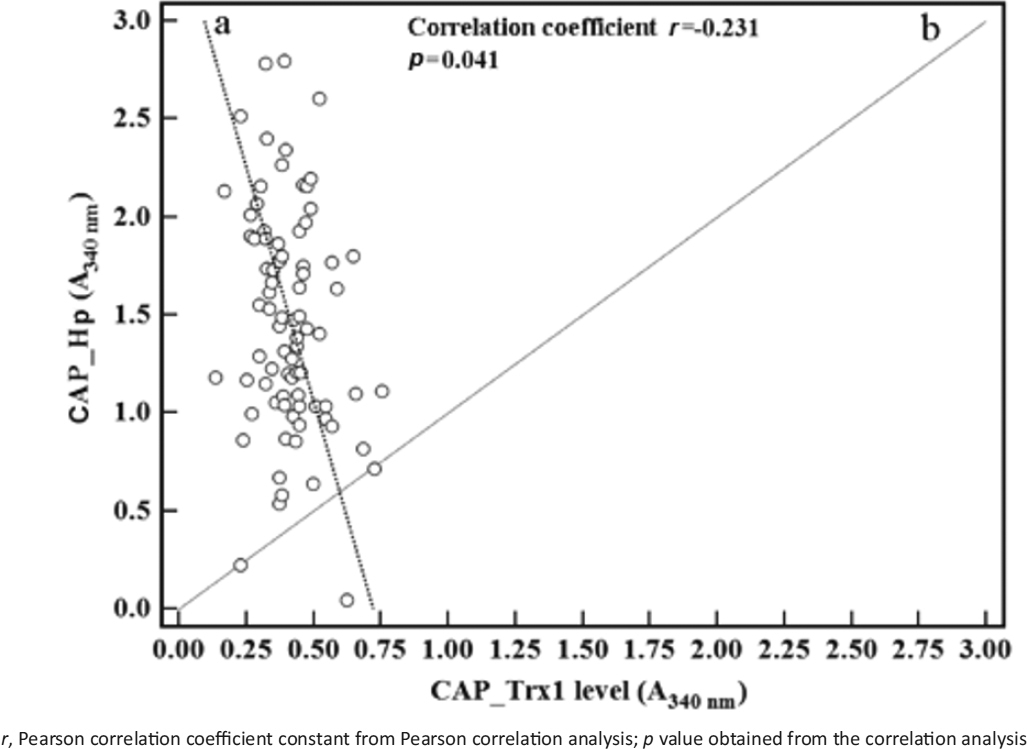
Correlation between the serum thioredoxin 1 (Trx1) and haptoglobin (Hp) levels in community-acquired pneumonia (CAP) patients. The individual mean serum levels (*n* = 3) in 80 patients with CAP (*n* = 40 for female and *n* = 40 for male) were depicted as a scatter diagram. The individual serum levels of Trx1 were displayed along the x-axis, and those of Hp in the corresponding patients are plotted along the y-axis. Lines “a” and “b” are reduced major axis line and line of equality, respectively.

As is understood from the data, Hp is a good marker for both male and female patients with CAP, whereas Trx1 exhibits a function similar to Hp in female patients. The characteristic difference between Hp and Trx1 is that the CAP group has a higher serum Hp level but a lower serum Trx1 level compared to the corresponding normal controls. To confirm these contrary results, the correlations between the Trx1 measurements and the Hp measurements of the CAP group were analysed, and the results are shown in Figure 3. The Trx1 measurements were found to be inversely proportional to the Hp measurements, (*r* = −0.231, *p* = 0.041), as seen in Figure 3. For testing for normality of the data, the D’Agostino-Pearson normality test was performed. The data sets for Trx1 and Hp levels were normally distributed (Trx1: *p* = 0.06; Hp: *p* = 0.30).

### 4. Discussion

This study showed that Trx1 can be used as a diagnostic marker for CAP and an effective companion diagnostic marker to Hp for lung cancer. Trx1 was found to be present in significantly lower concentrations in the serum of female patients with CAP than in the blood of normal females. In addition, the serum Trx1 levels in patients with cardiovascular diseases and lung cancer were measured to be significantly higher than those in normal controls. Furthermore, the serum level of Trx1 was observed to increase along with lung cancer progression. In contrast to Trx1, much higher serum levels of Hp were detected in CAP patients, as well as lung cancer patients without selectivity, compared to normal controls. The Trx1 measurements were found to be inversely proportional to the Hp measurements. Overall, the specifically remarkable decrease of Trx1 levels in female CAP patients suggests that the reduction may be caused by a female CAP-specific action and not by general oxidative stress and inflammatory actions. Trx1 was reported to be elevated in patients with various carcinomas and inflammatory diseases [8,12], which is consistent with our results showing significant increases of serum Trx1 levels in patients with lung cancer and cardiovascular disease.

On the basis of the results summarised above, Trx1 may be useful as a diagnostic marker for screening female patients with CAP; female CAP patients are characterised as having a lower serum Trx1 level compared to the controls. Monitoring Trx1 levels in the blood plasma may be useful for the diagnosis and follow-up of CAP. The serum level of Trx1 significantly decreases in the female patients compared to female controls with excellent specificity (89.8%) and selectivity (AUC = 0.877) for the female patients, regardless of whether they have been affected by cancer or non-cancer diseases. Trx1 has an advantage over Hp in selecting female patients with CAP because serum Hp levels increase in both female patients with lung cancer and CAP without specificity. Consequently, Trx1 can be used not only as a potential CAP marker in females but is also as an excellent companion diagnostic marker with Hp for discriminating CAP patients from females with high serum levels of Hp.

Considering a high serum level of Hp in both female patients with lung cancer or CAP and a significant decrease of serum Trx1 level in female patients with CAP, but the increasing tendency of serum Trx1 level as a function of the progress of lung carcinoma in the female patients, taken together, these observations suggested that serum Trx1 can be used as not only a novel pneumonia biomarker applicable to female patients, but also as a combinational marker with Hp for the specific diagnosis of female patients with lung carcinoma.

Trx1 serves not only as a reactive oxygen species detoxifier but also as a regulator of signal transduction pathways and peroxide responses [1,2]. Thus, a decreased serum level of Trx1 in CAP patients, especially in the female patients, remains unresolved. In addition to extracellular levels of Trx1 and Hp, it could be interesting to determine their intracellular levels in the paired tissues. Given that the reduction of the serum Trx1 level in CAP patients is not female-specific, the detailed *in vivo* function of serum Trx1 in CAP patients is worth further investigation. In fact under certain conditions, the expression of Trx1 or the secretion of Trx1 into extracellular fluid is either negatively or positively regulated, which can be explained by differences regarding cell types, cell conditions, or strength of stimulation.
